# Mitigating Contaminant-Driven Risks for the Safe Expansion of the Agricultural—Sanitation Circular Economy in an Urbanizing World

**DOI:** 10.1021/acsestwater.3c00803

**Published:** 2024-02-15

**Authors:** Laura J. Carter, Sarah Dennis, Katie Allen, Patrick McKenna, Xiaohui Chen, Tim J. Daniell, Barbara Evans, Jeremy S. Guest, Hongyan Guo, Stuart Kirk, Yong-Guan Zhu, Asif Reza Anik, Naqshe Zuhra, Steven A. Banwart

**Affiliations:** a School of Geography, University of Leeds, Leeds, LS2 9JT, U.K.; b Global Food and Environment Institute, University of Leeds, Leeds LS2 9JT, U.K.; c School of Earth and Environment, University of Leeds, Leeds LS2 9JT, U.K.; d School of Civil Engineering, University of Leeds, Leeds LS2 9JT, U.K.; e Molecular Microbiology: Biochemistry to Disease, School of Biosciences, The University of Sheffield, Sheffield S10 2TN, U.K.; f Department of Civil & Environmental Engineering, University of Illinois Urbana−Champaign, Urbana, Illinois 61801, United States; g State Key Laboratory of Pollution Control and Resource Reuse, School of the Environment, Nanjing University, Nanjing 210023, China; h The Schumacher Institute, The Create Centre, Bristol BS1 6XN, U.K.; i Research Center for Eco-environmental Sciences, Chinese Academy of Sciences, 18 Shuangqing Road, Beijing 100085, China; j Department of Agricultural Economics, Bangabandhu Sheikh Mujibur Rahman Agricultural University, Salna, Gazipur 1706, Bangladesh; k Institute of Soil and Environmental Sciences, University of Agriculture, Faisalabad 38000, Pakistan

## Abstract

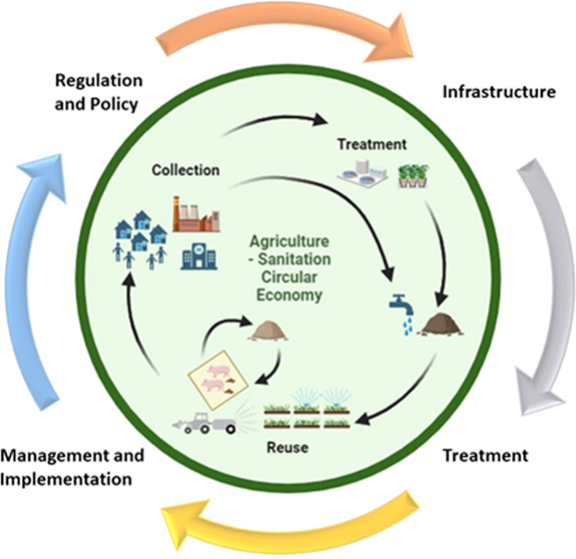

The widespread adoption of an agricultural circular economy requires the recovery of resources such as water, organic matter, and nutrients from livestock manure and sanitation. While this approach offers many benefits, we argue this is not without potential risks to human and environmental health that largely stem from the presence of contaminants in the recycled resources (e.g., pharmaceuticals, pathogens). We discuss context specific challenges and solutions across the three themes: (1) contaminant monitoring; (2) collection transport and treatment; and (3) regulation and policy. We advocate for the redesign of sanitary and agricultural management practices to enable safe resource reuse in a proportionate and effective way. In populous urban regions with access to sanitation provision, processes can be optimized using emergent technologies to maximize removal of contaminant from excreta prior to reuse. Comparatively, in regions with limited existing capacity for conveyance of excreta to centralized treatment facilities, we suggest efforts should focus on creation of collection facilities (e.g., pit latrines) and decentralized treatment options such as composting systems. Overall, circular economy approaches to sanitation and resource management offer a potential solution to a pressing challenge; however, to ensure this is done in a safe manner, contaminant risks must be mitigated.

## Introduction

Strengthening our food systems is essential. Demand for food is projected to double from 2010–2050 due to both increased human population and wealth.^1^ Rates of population growth are slowing but a projected increase to a global population of 9.7 billion^2^ in 2050 is expected to be accompanied by a quadrupling in the size of the global economy, a doubling in demand for energy and more than a 50% increase in the demand for clean water.^3^ This growing demand for primary resources of energy, land, water and food is taking place at a time when the impacts of climate change are expected to adversely impact resource provisioning.^4^

**Figure 1:**
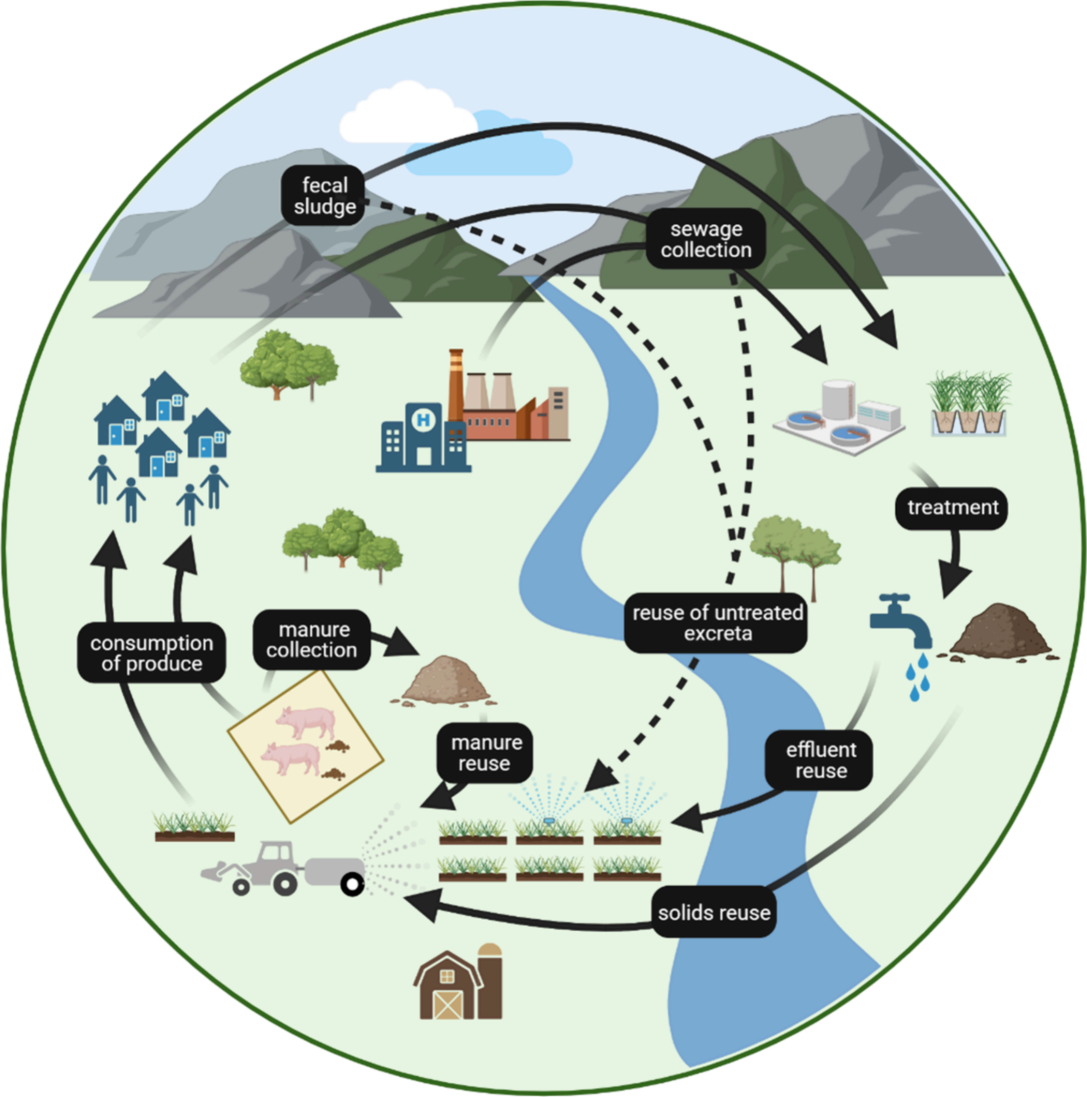
Concept of a sanitation–livestock circular economy to support agricultural production highlighting opportunities for the reuse of excreta. The solid lines represent the movement of excreta which is treated (municipal and onsite treatment). The dotted line shows the reuse of untreated excreta which is a practice we want to avoid to reduce the risks associated with contaminant exposure.

To meet this demand, the concept of a circular economy has been proposed to realize sustainable agricultural production; where waste does not exist and instead byproducts and materials, primarily from municipal and agricultural sources, are fed as raw materials back into agricultural systems to meet production demands for primary resources (Figure 1). The procurement of water, organic matter (C), and nutrients (N, P, K) from livestock manure and sanitation enables us close the resource loop with materials that would be otherwise disposed of, with financial and environmental costs, while offering many benefits to both the sanitation and agricultural sectors.^5,6^

The production of fertilizers, based on a volatile and finite mineral fertilizer supply chain, presents a very serious threat to food security and climate change. At a global scale, if universal sanitation coverage is achieved by 2030 and the proportion of untreated wastewater is halved, nutrient recovery from wastewater has the potential to replace 11% and 12% of projected agricultural N and K use, respectively.^7^ Estimates for P recovery range from 9 to 20% of that which is applied to agricultural production as a fertilizer,^7,8^ with greater potential for recovery in countries with high protein diets since this results in higher per capita rates of N and P excretion.^9^ N recovery also offers promise in terms of mitigating climate change by reducing the greenhouse gas emissions from industrial ammonia production and the associated manufacture of mineral fertilizer and its application to land.^10−12^ Recent estimates suggest that carbon recovered from sanitation could meet up to 12% of the annual C sequestration potential of the worlds’ agricultural land.^10^ Agriculture currently accounts 70% of global freshwater use,^13^ with the greatest demand originating from arid regions such as Northern Africa and Western Asia. Meanwhile more than 90% of global wastewater generated is discharged, untreated, into waterways.^14^ At the local scale, wastewater recovery for irrigation has the potential therefore to play a role in sustaining crop production, particularly in water stressed regions, where food production is heavily dependent on supplemental irrigation.

An agricultural circular economy is therefore an economic model that spans supply chains and sectors and redefines the process of product design, manufacturing, and consumption, thus opening up new, unexploited (secondary) markets for companies. Reuse loops have major advantages for the sanitation and agricultural sector. However, the concept of resource reuse is not new.

Despite a recent drive to implement sustainable restorative agricultural practices to meet agricultural demand (e.g., DEFRA^15,16^), circular economy principles to support food production have long underpinned traditional agricultural practices in many countries. Such practices exist in short-cycles (predominantly at the household or community level) as well as in long-cycles (for example, from networked urban sanitation or municipal solid waste collection systems). Sweden provides an example of long-cycle processes; 34% of the 200,000 tons of sewage sludge which are produced each year are spread on commercial farmland.^17^ Comparatively, China has a long tradition of recycling and composting of organic materials primarily in short cycles at the local level to support crop growth for local consumption. Wastewater from city-scale sewer systems is an important source of water and nutrients and is currently recycled for use in irrigation schemes worldwide, particularly, but not restricted to, countries in arid and semiarid regions.^18^ For example wastewater has been used >100 years to meet irrigation demands in the Mezquital valley, Mexico.^19^ However, since the early adoption of resource reuse we have experienced rapid industrialization, which has resulted in lasting transformations of business, economics, and the basic structure of society. An important byproduct of this was the manufacture of a widening range of chemicals that enter our waste streams with potential to be inadvertently released into the wider environment following resource reuse. Recent estimates suggest that over 350 000 chemicals and mixtures of chemicals have been registered for production and use,^20^ with a doubling of production capacity between 2000 and 2017.^21^ This trend shows no sign of slowing, with a total of 300 new active pharmaceutical ingredients alone expected to be launched by 2026, which is appreciably higher than the level seen on average during the past decade. The presence of emerging classes of contaminants with demonstrated (eco)toxicological effects therefore presents a new challenge^22^ which needs to be considered, among other stressors,^23^ when considering the potential for resource reuse to support future agricultural production. In this paper, we present an overview of the potential chemical and biological risks associated with resource reuse and then explore context specific challenges and solutions to mitigate risk across the three themes: (1) contaminant monitoring, (2) collection transport and treatment, and (3) regulation and policy.

### Contaminant Risks Associated with a Circular Economy to Support Agricultural Production

Contaminants inadvertently introduced into our environment have the potential to cause severe ecosystem and human health problems at different scales^21^ and as such have been recognized as one of the “planetary boundaries” (the environmental limits within which humanity can safely operate).^24^ Contaminants are of concern when these entities exhibit persistence, mobility across scales with widespread distribution, and accumulation in organisms and the environment. Advances in mass spectrometry offering lower limits of detection together with global monitoring campaigns have highlighted the ubiquitous presence of contaminants in resources destined for agricultural reuse including biosolids, treated wastewater and manures.^25^ Studies have reported the presence of physical contaminants, biological pollutants such as pathogens and a suite of chemical entities including mycotoxins, metals and emerging contaminants in human and farm excreta.^26,27^ Following application to land, these chemicals can remain present in soils, migrate to nearby water bodies, or be taken up by organisms (Figure 1). The accumulation of contaminants into species at the bottom of a food chain (e.g., crops), presents a wider ecosystem risk through food chain transfer, including a risk to human health following the consumption of contaminated produce which is the focus of our discussion.^28^

Metals are well-known environmental contaminants due to their toxicity, persistence in the environment and bioaccumulative nature.^29^ Metals have been reported to affect biochemical and physiological functions in plants and animals, and their uptake into species at the bottom of a food chain presents wider a risk via food chain transfer.^30^ Beyond metals, micronutrients such as Cu and Zn pose a threat to sustainable agriculture. Following use as additives to stimulate the growth of livestock and prevent disease their presence in animal manure can lead to the accumulation in the soil environment with excess Cu and Zn soil concentrations observed to inhibit plant growth and lower the uptake of other micronutrients such as Fe and Mn.^31^ As wastewater differs from freshwater in salinity, pH, and concentrations of suspended solids and dissolved organic matter, wastewater irrigation can change the soil’s physical, biological and chemical characteristics.^32^ For example, an increase in soil salinity can reduce plant growth,^33^ and long-term irrigation with wastewater has the potential to increase soil sodicity, which in turn reduces soil-structure stability.^34,35^

Organic contaminants are increasingly reported in sludges, manures, and wastewater which are then released into the environment following application to land.^26,36^ Although select compounds can be degraded or volatilized in soils, chemicals with high molecular weight can persist such as polychlorinated naphthalenes and perfluorinated surfactants and potentially affect soil microbial community and function.^37^ Comparatively, emerging contaminants such as pharmaceuticals have relatively short half-lives but their continual release, resulting in pseudopersistence, and their retained biological potency presents a risk to soil and plant health at environmentally relevant concentrations.^38^ The biological potency of antibiotics in particular can influence the structure and function of soil microbial communities and enhance the development and spread of antibiotic resistance genes (ARGs) thus contributing to the proliferation of antimicrobial resistance (AMR).^39^ The increased flow to the environment of ARGs from human and domestic livestock sources is of particular concern because of their coexistence with zoonotic pathogens and veterinary and medical antimicrobial compounds as selective agents in manure and sanitation sources.^40^ Organic fertilizer application also provides an important pathway for microplastics to enter into soil environment which has the potential to affect the development and health of plants while also influencing soil properties and ecosystem functioning.^41^ Moreover, microplastics can become hotspots for horizontal gene transfer of antibiotic resistance genes promoting the spread of antibiotic resistance between microbes.^42^ It is also important to note that sewage sludge and biosolids can be highly loaded with viruses of faecal origin and constitute potential repositories of pathogenic viruses.^43^ Therefore, the use of these materials also presents a route for biological contamination in the agricultural environment, ultimately threatening human health.^44^ For example, inadvertent exposure to pathogens has been shown to contribute to the burden of childhood norovirus, rotavirus and other enteric infections in environments where there is substantial faecal matter circulating.^45^ Chronic exposure to wastewaterborne pathogens is responsible for some of the most serious causes of infectious diseases in the world, 60 percent of diarrhea worldwide is associated with inadequate sanitation, and lack of water and sanitation together account for more than 5% of all deaths in children under five years old.^44^

## Context Specific Challenges

Circular approaches can help close the nutrient loop between the sanitation and agriculture sectors while addressing major global water, energy, and food security issues. In order to meet future agricultural nutrient and water demand, there is a need to scale up resource recovery and reuse.^5,7^ However, this will result in highly varied and situational challenges associated with supply and the presence of contaminants which are discussed below across the themes of (1) contaminant monitoring, (2) collection, transport, and treatment, and (3) regulation and policy.

### Contaminant Monitoring

The growing volume and diversity of contaminants (e.g., emerging contaminant) currently hinders authorities from adequately assessing and managing the associated risks to human health and the environment.^46^ Contaminant monitoring systems, such as quality assurance laboratories audits and certification systems, are often fragmented among sectors and stakeholders with limited opportunity to generate an integrated picture of cocontaminant exposure. In a global context, geographical differences underpin the types and concentrations of contaminants detected in the receiving environment. For example, recent genomic analysis of sewage from 101 countries revealed a relatively even two-way split of both bacteriomes and resistomes between Europe, central Asia, and North America and Sub-Saharan Africa, South Africa, Latin America, and the Caribbean. The highest total antibiotic resistant gene loads were on average observed in Sub-Saharan Africa.^47^ Similarly, higher concentrations of pharmaceuticals in wastewater are typically observed in low- to middle-income countries.^48^

Within country, differences also exist in terms of types and concentrations of contaminants typically detected. In rural areas much of the pollution originates from animal excreta (e.g., antibiotics) and agricultural chemicals (e.g., pesticides) in comparison to urban settings which are dominated by contaminants originating from industrial activity and household waste.^49^ However, these observed trends are built from limited data sets, and at both global and regional levels, we currently lack a comprehensive assessment of levels of contaminants across all aspects of resource capture, treatment, and reuse. While this information is crucial to identify contaminant hotspots or heightened levels of risk, the cost implications, and associated difficulties in monitoring at a farm level (e.g., access to analytical methods and equipment), make such efforts particularly challenging in a number of countries.

### Collection, Transport, and Treatment

Well-managed sewage and on-site fecal sludge management systems effectively separate people from human excreta and the associated contaminant risk and offer opportunities for resource capture for reuse. However, 40% of the global population do not receive this level of service.^50^ Where collection and transport infrastructure is absent, insufficient or poorly managed this may result in the inadvertent release of untreated material into the environment.^50^ It is also important to note even when collection and transport are in place, conveyance through a sewer does not necessarily result in the treatment of excreta as it is estimated that 30% and 90% of wastewater goes untreated in high and low income countries, respectively.^14^

Despite the overall profile of contamination being a factor of production and usage, pharmaceutical concentrations, for example, are often higher in settings where treatment coverage is incomplete and the quality of treatment is poor due to not being adequately financed and maintained. For example, a review of the occurrence of pharmaceuticals and personal care products in Indian water bodies found that levels of pharmaceuticals in wastewater treatment plant effluent were up to 40 times higher than reported elsewhere globally.^51^ Further research shows that treatment type and sewer connectivity strongly influenced the emission of rotavirus from wastewater in a comparison between the UK and Nigeria, where 100% and 25% of the urban population were connected by a sewer, respectively.^52^ Challenges associated with the large-scale treatment of excreta will only be exemplified in the future with most of the global population expansion predicted to take place in the Geopolitical South;^53^ with many regions projected to lack the financial resources and the provisioning of water resources to enable any substantial development of wastewater infrastructure.^10^

Similarly to the management of human excreta, manure management strategies are diverse; ranging from little to no treatment to more advanced technologies with varying impacts on contaminant concentrations.^54^ Composting has been shown to reduce the risks of pathogenic microorganisms, and while stockpiling and can create favorable conditions for the degradation of some organic chemicals such as veterinary antibiotics,^55,56^ contaminants such as heavy metals are persistent. Local, manure reuse loops where manure is moved onsite or to a neighboring farm (e.g., straw for muck) offer a means of minimizing contaminant exposure with respect to reducing risks associated with storage and transportation. However, following the intensification of farming systems and the separation of livestock and arable farms manure is often needed to move from areas of surplus to areas of deficit^57^ and therefore manure conveyance also presents similar risks to that of human excreta for example with respect to the dissemination of AMR between animal and agricultural niches.^58^ With such varied coverage and treatment quality of both human and livestock excreta, the application of a blanket circular system presents a significant challenge.

### Regulation and Policy

Where regulated, heavy metals and metalloids in human and animal excreta are typically within the limits set by policy standards to permit release into the environment.^59^ However, this does not preclude the possibility of soil heavy metal pollution following long-term repeated applications of wastewater and sludge which has been shown to exceed permitted limits for some heavy metals, such as Cu, Zn, and Cd in Zimbabwe, for example.^60^ Through the EU Water Framework Directive, Environmental Quality Standards are set for selected contaminants in wastewater including pesticides, PFAS, bisphenol A and pharmaceuticals (e.g., painkillers, anticonvulsants, or antibiotics) to ensure receiving water bodies achieve good chemical status.^61^ However, there are currently no legally binding obligations at the international level for chemicals of emerging concern in sewage sludge, wastewater or manure applied to land despite their widespread use and potential impacts on human and ecosystem health (e.g., AMR).^40^ It is also important to note that where regulation and policy enforcement are not in place, or not routinely enforced, raw sewage with higher contaminant loads is routinely released into the environment (e.g., via direct discharge or combined sewer overflows^62^).

## Solutions

**Figure 2:**
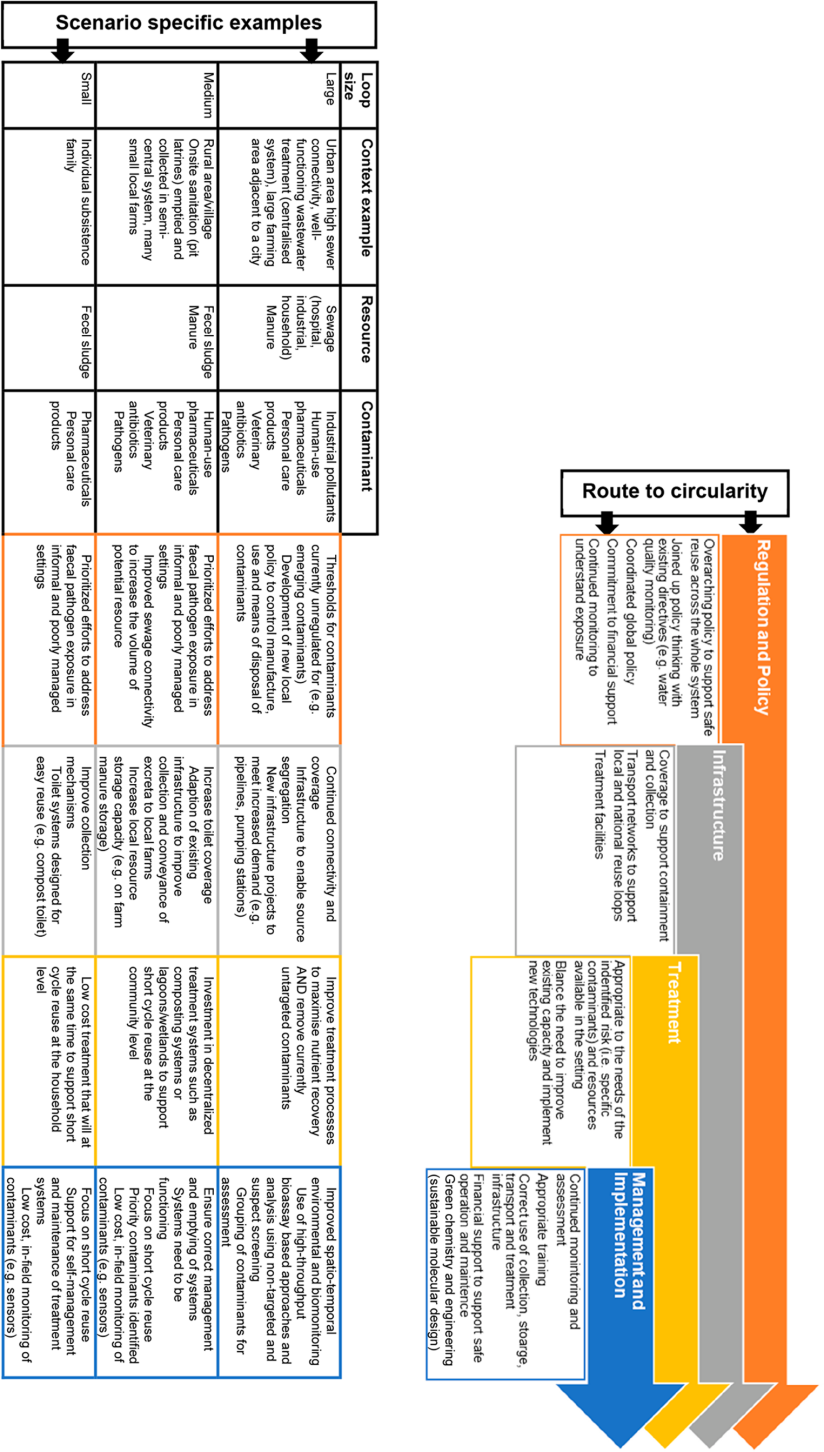
Steps to achieve a safe sanitation agriculture circular economy and scenario-specific examples detailing how to achieve this.

To enable the long-term, scaled-up, safe, and sustainable circular approach to agriculture and sanitation, many barriers need to be addressed, including the risk posed by contaminants. However, like the risk presented by chemical contaminants, potential mitigation measures are often influenced by a myriad of geographical and cultural challenges. Every opportunity for reuse is unique, owing to the highly variable nature of the supply and demand for resources and associated risks; we, therefore, advocate that there will not be a *“one-size fits all”* solution but herein discuss potential mitigation measures and prioritize key research needs (Figure 2).

### Contaminant Monitoring

There is currently a lack of consensus on which contaminants should be regulated in wastewaters and animal manure destined for land application. Until a comprehensive set of regulatory standards is developed, it remains unclear as to the extent we need to implement and improve treatment technologies to remove contaminants identified as being of potential concern. Further research is therefore first required to improve our understanding of the risks accounting for potential hazards (effects) and exposure in the environment to establish whether selected contaminants are dangerous to human health and therefore require a discharge limit. This is especially true for chemical contaminants for which we have limited data sets (e.g., emerging contaminants). However, it is not feasible to experimentally determine effect concentrations for all identified chemicals and for multicontaminant mixtures identified in the environment. Tackling groups of chemicals rather than single substances has the potential to accelerate chemical risk assessment;^63^ this together with high throughput methodologies and *in silico* efforts directed toward better understanding and prediction of the environmental fate of chemicals, would allow for systematic, “bottom-up” assessments of contaminants. These approaches coupled with suspect and nontarget screening of materials destined for reuse may help to ensure efforts are targeted toward chemicals, or chemical combinations, of most concern.^64^

It is also important to note that to effectively manage chemical risks within a country, it is important to address not only chemicals manufactured in, imported into, and/or used in the country but also those manufactured, used, disposed, and released in other countries. Joint action at a global level is therefore needed to deliver comprehensive environmental and biomonitoring programs, adhering to quality assurance and data harmonization criteria. Initiatives at a global level, such as UN level activity could promote dialogue and cooperation on this topic within countries (e.g., US EPA and USDA). The development of the Intergovernmental Science-Policy Panel on Chemicals, Waste, and Pollution Prevention also offers promise with respect to global waste management strategies.

### Collection, Transport, and Treatment

Following the identification of particularly risky inorganic and organic contaminants,^45^ the adaption of existing infrastructure (e.g., collection mechanisms) together with new infrastructure projects (e.g., pipelines, pumping stations and treatment methods) will be needed to prevent the spread of contamination and enable safe reuse. This will result in a cascade of benefits beyond meeting the agricultural demand for resources. Any changes to human or animal excreta management strategies must be optimized to meet the needs of the community. In populous urban regions with access to sanitation provision, processes can be optimized using emergent technologies such as electrochemical stripping,^65^ to reduce the loss of valuable nutrients (e.g., N) and tailored to remove contaminants previously not considered in the design of traditional treatment facilities (e.g., pharmaceuticals). This would create a high-quality product to meet local fertilizer requirements with the potential to be transported further afield.^66^ However, given that most of the projected population increase in urban areas is expected to be highly concentrated in a handful of countries, with limited existing capacity for conveyance of excreta to centralized treatment facilities, (e.g., India, China and Nigeria will account for 35% of the projected growth of the world’s urban population between 2018 and 2050^2^), investment in on-site sanitation is needed to avoid the high upfront capital costs of sewers and need to retrospectively construct sewers through existing homes and settlements. Efforts should therefore focus on creation of excreta collection facilities (e.g., pit latrines) and decentralized treatment options such as composting systems^67^ or “sewer mining”^68^ to support short cycle reuse of nutrients and water at the household/community level.^69^ This may also offer several advantages, including relatively lower transport and processing costs of dewatered materials.

Treatment may need to be tailored toward specific reuse streams and respond to the types of contaminants present in the material. For example, sewage usually contains a high incidence of contaminants from medical and industrial sources, and so controlling for these is paramount. Conversely, many decentralized systems exclude these waste streams, and so these contaminants are of lesser concern; however, the pathogen concentration may be higher if there is no dilution with flushwater. The smaller the scale of the collection and reuse loop, the easier it is to control the types of contaminants which are entering the reuse stream. In theory designing effective source segregation of different waste streams could eliminate the need to treat for certain contaminants completely, enabling more efficient treatment and safer reuse.^70^ For pathogens, highly mechanized wastewater treatment plants are relatively successful in decreasing pathogen load but targeted efforts are needed to address fecal pathogen exposure in informal and poorly managed settings and following improper reuse of wastewater effluents and solids. Low cost options such as composting, drying, or long-term storage offer viable alternatives for high pathogen removal too in resource limited settings.^71^

Ultimately, the level of treatment will depend on the setting and the desired product while also meeting the requirements of different crops, soil types, and climate. In line with practices currently ongoing in the US, highest quality treated wastewater with minimal contaminant loading could be used to irrigate food crops whereas partially- or secondary-treated water could be used for landscaping irrigation or surface irrigation of nonedible portions of food crops (e.g., nut trees).^72^ In China, vegetables are typically cooked which may provide a cultural in-home mechanism to more safely manage risks associated with small scale local reuse loops (e.g., manure application) in comparison to semiarid countries such as Israel where uncooked leafy vegetables are grown under large-scale wastewater irrigation schemes and may require lower contaminant levels.

### Regulation and Policy

Policy surrounding the reuse of materials to support agricultural production requires the setting of appropriate quality standards for resources that account for potential health risks. Across the globe many separate policies and regulations exist to prevent pollution from sanitation and agricultural resource streams (e.g., EU Nitrates Directive (91/676/EEC); Soil Pollution Prevention and Control Action Plan in China^73^). In the US, the reuse of biosolids is underpinned by regulation with specifies limit values for select chemicals and pathogens and direct use on agricultural land is determined according to a classification system.^74^ Country specific policies therefore exist upon which a future circular economy policy for safe global reuse can be built. However, in response to scientific advances, new policies and harmonized classification schemes will need to be developed to address the control the manufacture, use, and disposal of contaminants not covered by existing regulation but where risks are identified (e.g., pharmaceuticals, microplastics). Policy to reduce antibiotic use in livestock farming is an example of a successful initiative which has reduced antibiotic pollution in the wider environment.^75^ Efforts are now needed to tackle the overconsumption of all chemicals and reduce exposure via source control. Integration of sustainable molecular design, a concept directly stemming from green chemistry principles 4 and 10,^76^ into the development of new chemicals will also lessen the burden of chemical exposure.

In addition to setting regulatory standards, a policy to support the continuous development of sewer infrastructure and on-site sanitation for the safe collection and treatment of resources is needed (Figure 2). Accordingly, a pricing scheme that incentivizes efficient reuse of “waste” resources should be implemented, considering public perception and the productivity and supply costs (including treatment) of these materials relative to existing resources such as synthetic fertilizer. As has been discussed, a *“one size fits all”* approach to circularity is not possible given the regional and cultural challenges many countries face. In settings where it is not possible to implement comprehensive wastewater collection and treatment programs, near-term risk management and interim solutions are needed. This could include a combination of source control, and farm-level and postharvest measures, such as producing only industrial or nonedible crops in contaminated soils. Despite identified regional differences, we need to ensure that new policy frameworks for pollution control are collectively coherent and in line with existing policies for resource reuse. Policies need to be underpinned by appropriate financial support to enhance action across the science-policy interface to link policy thinking and improvements across the farming and business sectors.

## Financial Support Can Enable Timely Action

To increase the acceptance of excreta-based fertilizers, this needs to be financially attractive and as simple as possible. Increasing capacity for safe reuse will also require long-term planning and significant shifts in investments in key sectors linked to the presence of contaminants, including collection, storage, treatment, and transportation of resources. Because of the weak link between the benefits of subsequent safe reuse and enhanced treatment to remove contaminants, many countries seem unwilling to bear the financial burden of investment in operation and maintenance of treatment systems to reduce potential chemical and biological hazards.^77^ However, examples of successful operations to achieve reuse at a scale do exist. Following $750 million of investment Israel now operates 67 large wastewater treatment facilities and a network of pipes to enable 90% of treated wastewater to move to agricultural areas for irrigation.^78^ Therefore, where centralized sanitation systems exist, strategic investments will first be required to improve transport networks, treatment, and storage infrastructure for materials destined for land application. At a local level, any changes to agricultural management practices will also come at a direct cost to the farmer, which will need to be subsidized to ensure equity and affordability and long-term compliance such as financial support for the implementation of an irrigation network or manure/slurry collection and storage.

However, as highlighted above, country specific challenges exist, and the implementation of consistent regulation and policy does not occur equitably among countries around the world. For example, it is unrealistic to expect all countries to secure investment at the scale needed to deliver centralized wastewater treatment as achieved in Israel. Changes to the treatment of excreta will therefore need to be met by diverse investments that are sustainable and appropriate to the needs of the country, including international aid and targeted philanthropic investment which to date has largely focused on communicable diseases and not contaminant threats to public health and the environment. Where governments are pressed with struggling economies and many competing priorities, investment needs to be directed toward supporting sustainable small scale local reuse loops and innovative technologies to facilitate safe resource recovery (Figure 2). For example, compost toilets and source separation of feces and urine together with initiatives to reduce chemical consumption (i.e., antibiotic prescribing) offer solutions reduce risks associated with reuse and have many far reaching benefits (e.g., improved water and sanitation). Beyond financial and technological initiatives, we should work to make the sector more attractive to investors and more accountable to the public; sanitation and agricultural sectors need to improve their technical and financial efficiency, and the administrative, governance, and regulatory regimes overseeing this need to be more transparent and accountable.

Where costs are incurred these could at least be partially offset by the value of the resources captured using a model similar to that already practiced for energy recovery from wastewater treatment processes.^79^ However, this raises a further set of challenges in quantifying the financial value of the resources; accounting for cost savings made from not having to pay for waste disposal, from the costs of climate impacts of fertilizer production and use and from the purchase of traditional agrochemicals. Although progress has been made in this area a lack of standardized and comparable data and metrics makes it a challenge to calculate a risk/return ratio for an agriculture circular economy.^80^ Improved economic methods or tools that better capture the value of ecosystem services (e.g., using systems approaches and natural capital accounting), or that places a higher intrinsic value on the environment and sustained provisioning of natural resources, are needed to create more accurate and realistic cost-benefit assessments.^81^ If managed appropriately and targeted to key areas of need, financial support offers a means of catalyzing effective communication and national coordination and stimulates entrepreneurial activity (e.g., novel treatment technologies) and experimentation to deliver a safe scaled up agricultural circular economy.

## Conclusions

A sustainable economy should be both circular and smart, and can become a source of renewable, water, and nutrients enabling farmers to “act locally” to meet farming demands while also addressing global ecosystem challenges such as climate change, water security, pollution, and topsoil loss. Resource reuse is much cheaper than alternatives such as desalination and can enhance an economy’s ability to address the growing imbalance between resource supply and demand.^22^ Improperly managed excreta present a risk to human and ecosystem health and create negative societal costs. However, if treated properly and contaminant risks are managed, it becomes a precious resource.

Nevertheless, it is important to highlight that even when all barriers to reuse have been overcome, and any health risks arising from contaminants are mitigated for, risk perception remains a significant barrier to social acceptance of the reuse of resources in agriculture, with concerns in particular around the use of human excreta derived materials.^79^ Acceptance of resource reuse is limited by information and availability and therefore interventions are needed that focus on community specific social and behavior change communication that embraces coproduction of change with local farming organizations and members of the community.^82^ As a global community, we need to re-envisage what “waste” is to increase public acceptability.

To support such a transformational shift in both the agricultural and sanitation sectors, research groups and industry must work with regulators to address the identified risks. Moreover, a transdisciplinary understanding of the proposed transition to circularity that accounts for multistakeholder perspectives is required to achieve a “just” transition to a sustainable agricultural food system for all. This will require focused research efforts to help plan for, and underpin, the transition and for the resulting new knowledge to be made available and accessible for use by all interested parties, while acknowledging the country and regional specific challenges that exist. Relevant research programmes and policy commitments to date, such as the European Commission Green–Deal and related assessments by the European Environment Agency,^83^ pave the way for this but continued efforts are needed.

Overall, circular economy approaches to sanitation and resource management offer a potential solution to a pressing challenge; there is a clear opportunity for the agricultural sector to rethink how it does business and to take the next steps to achieve the 2030 Sustainable Development Agenda. However, this will require us to redesign sanitary and agricultural management practices in a single holistic, circular model, to ensure this is done in a safe manner acknowledging the potential risks associated with the presence of contaminants in reuse resources.

## Dedication

With this paper we as a team honour the scholarly leadership of critical zone researcher Professor Steven Banwart, whose recent and sudden passing we grieve. Steve was an expert in soil and water resource protection for food security. Steve’s legacy within our research community will be to continue global leadership in tackling global challenges.
